# Impact of early identification of patients meeting testing criteria for *Clostridioides difficile* on standard infection ratios

**DOI:** 10.1017/ash.2023.279

**Published:** 2023-09-29

**Authors:** Brad Krier, Eric Gomez-Urena, Kristin Schultz, Ashley Brooks

## Abstract

**Background:** *Clostridioides difficile* infection (CDI) poses a health burden to patients and a financial burden to hospital systems. Timely identification of CDI patients can reduce the impacts by allowing for prompt treatment and ensuring that proper isolation precautions are in place to prevent spread. It also ensures correct CDI event categorization according to the NHSN. Community-onset (CO) CDI cases are tested on or prior to hospital day 3, and hospital-onset (HO) CDI are tested on or after hospital day 4. The objective of this study was to determine the effectiveness of utilizing an electronic health record (EHR) report to reduce CDI standard infection ratios (SIRs) by identifying potential CDI cases prior to hospital day 4. **Methods:** From August of 2021 to September 2022, an EHR report was implemented in a 5-hospital healthcare system in the Midwest to identify patients with 3 or more type 6 or 7 stools in a 24-hour period based on Bristol stool chart classification. All inpatients with 3 or more type 6 or 7 stools in 24 hours without an active order for a *Clostridioides difficile* test were listed. Patients with a laxative in the previous 48 hours, tube feedings without fever or leukocytosis, or a known cause of diarrhea were excluded. The attending provider of the patients meeting criteria were notified with a recommendation to test for *C. difficile* or provide alternative reason for symptoms. **Results:** In total, 26 patients were tested for *C. difficile* using polymerase chain reaction testing. Of those tested, 5 (19.2%) tested positive for *C. difficile*. There were 13 HO-CDI cases for the healthcare system during this period, for an SIR of 0.351. If the early identified cases were not identified until after hospital day 3, the SIR had the potential to have been 35.6% greater at 0.476. **Conclusions:** We were able to identify 5 CDI cases prior to hospital day 4 using an early identification report during this 13-month period. Although these cases may have been identified without the use of the EHR report, we were able to obtain a timely CDI diagnosis, potentially limiting the spread of *C. difficile* and preventing an increase in the CDI SIR by 35.6%. An EHR report to identify patients meeting *C. difficile* testing criteria may be an effective way to identify CO-CDI prior to HD 4 and thus reduce CDI SIR

**Disclosures:** None

